# Primary Sclerosing Cholangitis in Crohn’s Disease: An Atypical Complication

**DOI:** 10.7759/cureus.14964

**Published:** 2021-05-11

**Authors:** Mindy C Ward, Blake Studer, Ian Nora, Nathan Seepaulsing, Charles Loewe

**Affiliations:** 1 Florida State University College of Medicine Internal Medicine Residency, Sarasota Memorial Hospital, Sarasota, USA

**Keywords:** crohn’s disease (cd), psc

## Abstract

Crohn’s disease (CD) is a type of inflammatory bowel disease (IBD) that affects thousands of Americans, and it is commonly found in individuals aged between 20-30 years. Patients often present with abdominal pain and describe concerns of diarrhea, bloating, and weight loss. In this report, we discuss the case of a 21-year-old man who presented with diarrhea and intermittent hematochezia. He was admitted for a suspected lower gastrointestinal (GI) bleed. An abdominal CT scan demonstrated pancolitis with a mildly distended gallbladder. Subsequent sigmoidoscopy revealed a diagnosis of CD, which was confirmed with a biopsy. Following steroid therapy, the patient reported symptomatic improvement, although his alkaline phosphatase (ALP) levels continued to increase. Magnetic resonance cholangiopancreatography and endoscopic retrograde cholangiopancreatography (ERCP) revealed biliary strictures, which were suggestive of primary sclerosing cholangitis (PSC). This case highlights the importance of not ruling out CD in patients with PSC and understanding the differential clinical outcomes in patients with PSC with ulcerative colitis (UC) compared to those with CD. These differences include variations in colorectal carcinoma risk and severity of symptoms.

## Introduction

Crohn’s disease (CD) affects approximately 785,000 Americans and has a bimodal distribution, with disease onset frequently occurring between the ages of 20 and 30 years, and then later around the age of 50 [1]. Patients with CD generally experience fever, malaise, weight loss, abdominal pain, and non-grossly bloody diarrhea [2]. CD is classically associated with areas of transmural inflammation that can occur at any point in the gastrointestinal (GI) tract from the mouth to the anus, known as “skip lesions” [3]. However, the inflammation most commonly resides in the terminal ileum [4]. Also, microscopic noncaseating granulomatous pathology is commonly detected in CD [5]. While CD’s precise pathogenesis is unknown, it is believed to be due to an abnormal immune response to the gut flora [6]. Typical complications include perianal disease, bowel stricture, fistulas, abscess, and malabsorption [7]. One complication of inflammatory bowel disease (IBD), commonly associated with ulcerative colitis (UC), is primary sclerosing cholangitis (PSC) [8]. A hallmark of PSC is fibrosis and stricturing of the biliary system secondary to intra- and extrahepatic bile duct inflammation [9]. PSC’s clinical features can include fever, fatigue, pruritus, jaundice, right upper quadrant pain, and possibly end-stage liver disease [8,9]. We report a case of a young man who presented with diarrhea and was found to have CD with the less frequently associated PSC.

## Case presentation

A 21-year-old man with no significant past medical history presented to the emergency department (ED) with concerns of diarrhea that had started one month prior, and had worsened one week before the presentation. Initially, he had experienced an episode of diarrhea every other day, and then three to five episodes of watery brown diarrhea on a daily basis. Two days before admission, he had passed large amounts of bright red blood per rectum with associated non-bloody emesis. Over the previous month, he had lost weight, although he was unable to quantify the weight loss. He had no past medical history, had not undergone any surgeries, and not taken any medication besides occasional loratadine for seasonal allergies. He denied any relevant family history and was an occasional alcohol drinker, non-smoker, and had no history of illicit drug use. He had visited the ED only once previously, for diarrhea following a course of antibiotics for a nonhealing mouth ulcer.

**Figure 1:**
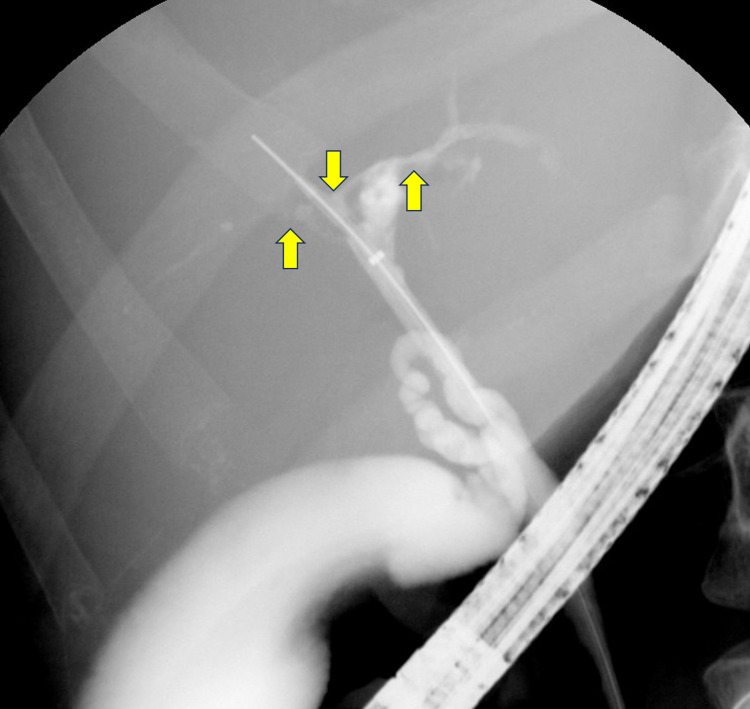
ERCP fluoroscopy indicating PSC (arrows) ERCP: endoscopic retrograde cholangiopancreatography; PSC: primary sclerosing cholangitis

**Figure 2:**
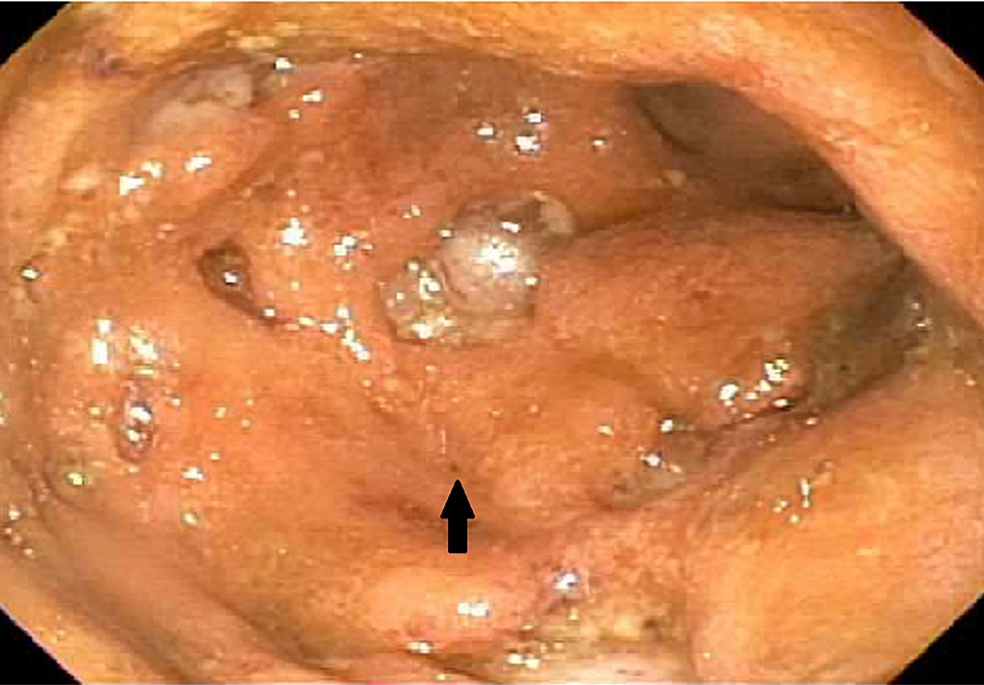
Descending colon The findings include ulcers larger than 2 cm, greater than 30% ulcerated surfaces, with more than 75% of surfaces affected

**Figure 3:**
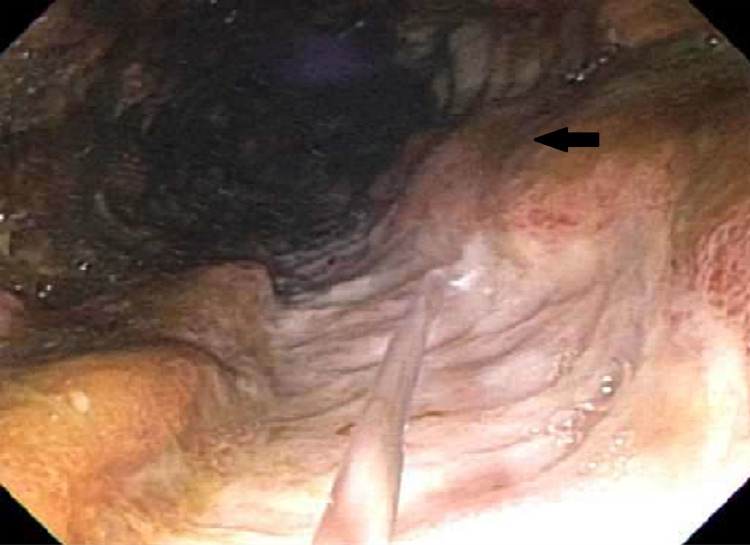
Large ulcer in descending colon (arrow)

Upon presentation, the patient was tachycardic and tachypneic. Notable vital signs were a heart rate of 132 beats per minute and blood pressure of 87/72 mmHg. Oxygen saturation and body temperature were within normal limits. His laboratory investigation revealed a hemoglobin of 7.6 g/dL, leukocytosis (white blood cell count of 19.6 x 10^9^/L), platelets elevated at 882 x 10^9^/L, and elevated alkaline phosphatase (ALP) at 430 U/L. His fecal immunochemical test was positive for blood. He was given a bolus of Ringer’s lactate solution and started on empiric antibiotics. He was subsequently admitted for the management of acute blood loss anemia secondary to GI bleed and was transfused a total of three units of packed red blood cells. CT of his abdomen revealed pancolitis with a mildly distended gallbladder. Flexible sigmoidoscopy was performed on his first day of admission, and pathology revealed CD. He received three days of intravenous methylprednisolone, which was subsequently switched to oral prednisone 60 mg daily. After the initiation of the steroid regimen, he reported no episodes of bloody diarrhea. His hemoglobin eventually stabilized to be between 9.9 and 10.6 g/dL. His hemoglobin and hematocrit levels were monitored closely throughout the admission period. Liver function studies revealed persistently elevated ALP, and his gamma-glutamyl transferase (GGT) level was also elevated. Magnetic resonance cholangiopancreatography revealed a long segment stricture of the common bile duct. Endoscopic retrograde cholangiopancreatography (ERCP) on day four revealed that one-third of his common bile duct was dilated; mucus and sludge were present, as well as severe diffuse strictures, which were concerning for PSC (Figure 1). Biopsies were taken during this procedure for cytology, and the patient was informed of these findings and the pathology results from the previous flexible sigmoidoscopy (Figures 2, 3). He was educated on the importance of close follow-up with an outpatient GI physician.

By the morning of his discharge, his hemoglobin had stabilized, and he was no longer experiencing bloody diarrhea, nausea/vomiting, or abdominal pain. The patient was instructed to follow up in one week to initiate ustekinumab therapy, and he was discharged with a long tapering dose of oral prednisone.

## Discussion

PSC is typically thought to be associated with UC; however, this is not ubiquitous as a review of the literature has indicated that PSC is associated with IBD only in 70-80% of cases, while the remaining 20-30% cases do not have a concomitant diagnosis of IBD. The prevalence of IBD in PSC may be even higher, as IBD diagnosis depends on the quality and intensity of endoscopic screening and biopsies obtained. Of the PSC cases associated with IBD, approximately 80% are classified as having concomitant UC, while approximately 10% have CD, and an additional 10% have indeterminate colitis. Of all patients with IBD, up to 8% of those with UC are likely to harbor PSC, while only 3% of patients with CD develop this complication [10].

As for clinical manifestations and prognosis, patients who have PSC in the setting of UC typically exhibit an earlier onset of colitis compared to those with IBD only. However, the intensity of colitis is typically mild or even asymptomatic in these patients, with few and sporadic episodes of rectal bleeding [11]. Patients with PSC and UC are at an increased risk of colorectal cancer (CRC). This patient population evinces up to a 50% increase in the absolute risk of contracting CRC at 25 years after diagnosis, while the patients with UC alone have a 10% risk [12]. However, this association has not been established in patients with CD and PSC. A 2016 meta-analysis of 16 observational studies confirmed that those with PSC in IBD had an increased CRC risk. When these data were segmented among IBD subtypes of CD and UC, researchers observed that PSC in CD was not associated with an increased risk of CRC. It should be noted that this meta-analysis only included three studies that included patients with both CD and PSC [13]. However, a 2014 study compared the outcomes of patients with PSC and UC or CD and found that those with CD and PSC had a lower risk of colonic neoplasia and colectomy [14]. However, this study is also limited as it only included 50 patients with CD compared to 223 with UC. Studies with a larger CD patient population need to be conducted to further examine the association between PSC in CD and colonic neoplasia risk.

## Conclusions

We discussed a case of a 21-year-old man with CD who was found to have PSC. While PSC is commonly associated with UC, physicians should always keep CD in the differential to avoid a missed diagnosis. This case highlights the importance of differentiating between IBD classes in patients with PSC as there are differences in management, symptom severity, and colorectal carcinoma risk.
